# Negative association of serum neurofilament light chain with estimated glomerular filtration rate levels and the impact of gender

**DOI:** 10.3389/fneur.2024.1457984

**Published:** 2024-09-11

**Authors:** Hongyan Peng, Zhuoxin Liang, Bolun Huang, Senxiong Zhang, Yiyu Yang

**Affiliations:** ^1^Department of Pediatric Intensive Care Unit, Guangzhou Women and Children’s Medical Center, Guangzhou Medical University, Guangzhou, Guangdong, China; ^2^Department of Intensive Care Medicine, Liuzhou Hospital of Affiliated Guangzhou Women and Children’s Medical Center, Liuzhou, Guangxi, China

**Keywords:** neurofilament light chain, estimated glomerular filtration rate, gender, kidney, brain

## Abstract

**Background:**

The relationship between kidney function and brain function is complex and poorly understood. This study aims to investigate the association between serum neurofilament light chain (sNfL) and levels of estimated glomerular filtration rate (eGFR), offering new insights into their interactions.

**Methods:**

Data from the national health and nutrition examination survey (NHANES) in 2013–2014, linked with national death index records, were used. Participants who met specific criteria were analyzed. Baseline characteristics were stratified by tertiles of sNfL levels and compared using weighted Kruskal-Wallis and chi-square tests. Weighted linear regression models, both unadjusted and adjusted, evaluated the relationship between log sNfL and eGFR. Subgroup and interaction analyses validated the findings. Restricted cubic spline, scatter plots, and Spearman correlation confirmed the relationship between log sNfL and eGFR.

**Results:**

A total of 2,038 eligible participants were included. Higher sNfL levels were significantly associated with lower eGFR (*p* < 0.01). The highest sNfL tertile had a significantly higher mortality rate (*p* < 0.01). Fully adjusted multivariable weighted linear regression showed a significant negative correlation between log sNfL and eGFR (per 10-unit increase; *β* = −0.07, 95% CI: −0.10 to −0.04, *p* < 0.01). Subgroup analyses consistently supported this negative correlation (*p* < 0.01). Interaction analysis revealed a significant gender difference (*p* = 0.032), with males showing a − 0.06 (−0.09, −0.04) decrease and females a − 0.07 (−0.11, −0.04) decrease in log sNfL per 10-unit increase in eGFR. Restricted cubic spline confirmed a linear relationship (*p*-non-linear = 0.121), and the Spearman correlation coefficient was −0.45. Females had slightly lower log sNfL levels compared to males at equivalent eGFR levels.

**Conclusion:**

A significant negative correlation was found between log sNfL and eGFR levels. Gender influenced the degree of this negative association. Further research is needed to validate these findings and elucidate their underlying mechanisms.

## Introduction

Neurofilaments is a protein widely expressed in neuronal cells, comprising light, medium, and heavy chains. Neurofilament light chain (NfL) is a subunit of neurofilaments. It plays a critical role in maintaining neuronal structural stability and function, making it a specific biomarker within the central nervous system (1). Increased release of NfL occurred during neuronal axon inflammation, neurodegeneration, trauma, or ischemic damage. NfL was detectable in both cerebrospinal fluid (CSF) and blood, offering sensitivity in assessing neurological injury and disease progression (2–4). Consequently, serum neurofilament light chain (sNfL) holds promise as an essential tool in future neurological disorder management.

Estimated glomerular filtration rate (eGFR) serves as a vital indicator in assessing kidney function health, widely used in clinical practice. It is computed based on factors including serum creatinine levels, age, and gender, directly reflecting kidney efficiency in waste and fluid clearance (5). Precise and regular eGFR monitoring facilitates early identification and intervention of declining kidney function, effectively slowing chronic kidney disease (CKD) advancement and aiding in predicting overall mortality (6).

In recent years, there has been growing interest in the complex interplay between kidney function and brain function (7, 8). Studies by Sun et al. suggested that declining kidney function correlated with abnormal Alzheimer’s disease biomarkers and cognitive impairment, implying a potential role of kidney function in the pathogenesis of AD (9). Research by Chen et al. highlighted the close correlation between kidney function and cortical brain structure and function (10). The interaction between kidney and brain function involved various mechanisms such as neuroendocrine disruption, oxidative stress, chronic inflammation, and cerebral vascular damage, which could affect brain structure and function through multiple pathways (11). In recent years, several studies have identified kidney dysfunction as a risk factor for elevated sNfL levels in patients with multiple sclerosis and atrial fibrillation (12–14), and community-based studies also showed a negative correlation between sNfL and eGFR levels. However, these studies often focused on specific diseases or smaller, older populations, limiting the generalizability of their findings. This study utilized comprehensive data from all participants in the 2013–2014 NHANES database to explore the association between sNfL and eGFR levels, providing initial support for understanding the potential link between kidney and brain function, and facilitating a more accurate assessment of sNfL’s significance in neurologic disorders.

## Methods

### Study population

Our study data were derived from the national health and nutrition examination survey (NHANES), which employed a complex, stratified, multistage probability sampling design to ensure representative participant selection. All participants provided written informed consent, and the study protocol was approved by the NCHS Ethics Review Board. Participants from the NHANES 2013–2014 cycle were included as this was the period with available serum sNfL measurements. According to information from the NHANES website,^1^ in the 2013–2014 cycle, only participants aged 20–75 who had consented to have their samples stored for future research and who had surplus or pristine serum samples were eligible for sNfL measurement. Complete data on gender, age, ethnicity, body mass index (BMI), education, smoking status, alcohol consumption, hypertension, diabetes, cardiovascular and cerebrovascular related diseases (CVD), sNfL, and kidney-related indicators (including serum creatinine, blood urea nitrogen, uric acid, urine albumin, and urine creatinine) were required for inclusion.

### Calculation and grouping of sNfL

sNfL measurements were conducted using an innovative high-throughput acridine ester (AE) immunoassay, integrated into the Atellica platform by Siemens Healthineers. Serum samples were initially treated with AE-labeled antibodies that bound to the sNfL antigen. Subsequently, the mixture was combined with paramagnetic particles (PMP) coated with a capture antibody, forming an antigenic complex of AE-labeled antibody and PMP. After isolating and removing unbound AE-labeled antibodies, acids and bases were added to initiate chemiluminescence, and the emitted light was quantified. Each phase of this process was fully automated using the Atellica immunoassay system. Detailed laboratory procedure guides were available on the NHANES website. Based on log sNfL values, participants were divided into tertiles: T1, T2, and T3, from lowest to highest.

### Calculation and grouping of eGFR

The eGFR was calculated using the CKD-EpI 2021 equation (5, 15), which took into account age, gender and serum creatinine levels, as detailed in the literature. Serum creatinine measurement was performed using the Jaffe rate method on the DxC800 modular chemistry analyzer. In this procedure, a precise volume of the sample was introduced into a reaction vessel containing an alkaline picrate solution. Absorbance measurements were taken at 520 nm between 19 and 25 s after the sample was injected. The creatinine in the sample reacted with the reagent to form a red-colored complex. The rate of absorbance change was directly proportional to the creatinine concentration in the sample. Based on eGFR values, participants were categorized into three groups: low (<60 mL/min/1.73 m^2^), medium (60–89 mL/min/1.73 m^2^), and high (≥90 mL/min/1.73 m^2^).

### Assessment of covariates and mortality data

To minimize confounding effects on study outcomes, covariates were identified based on the literature. These variables included gender, age, BMI, ethnicity (categorized as non-Hispanic White, non-Hispanic Black, or other), and education level (classified as less than high school, high school or above). Alcohol consumption was defined as moderate or more if females consumed at least 1 drink per day or males consumed at least 2 drinks per day; otherwise, it was categorized as light or less (16). Smoking status was categorized as never smoked (<100 cigarettes in a lifetime), former smoker (≥100 cigarettes smoked but currently not smoking), and current smoker (currently smoking). Self-reported chronic conditions included hypertension, CVD (including angina, congestive heart failure, coronary heart disease, and stroke). The diagnosis of diabetes mellitus included (17): (1) self-reported diagnosis of diabetes; (2) use of antidiabetic medications; (3) hemoglobin A1c (HbA1c) level ≥ 6.5%; (4) fasting blood glucose ≥126 mg/dL. Additionally, kidney-related indicators collected included blood urea nitrogen, serum creatinine, serum uric acid, urine albumin, urine creatinine, and urine albumin-to-creatinine ratio. Mortality information obtained from death certificates in the national death index was linked to NHANES using participant sequence numbers common to both datasets. Specifically, this study utilized the publicly available linked mortality files from NHANES 2013–2014, providing follow-up duration and causes of death for adult participants up to December 31, 2019.

### Statistical analysis

We applied WTSSNH2Y as the sample weight to adjust for the complex multistage probability sampling design and analyzed sNfL levels. All analyses were conducted in line with NHANES guidelines, taking into account sample weights, clustering, and stratification. For more details on sample weight usage and other analytical considerations, please refer to the online tutorials available (18).^2^ Data for continuous variables were presented as weighted means ± standard deviations (SD) or median and interquartile range (IQR), while categorical variables were shown as unweighted frequencies (weighted percentages). The *p*-value, representing the differences among the sNFL tertiles, was calculated by applying a weighted Kruskal-Wallis test for continuous variables and a weighted chi-square test for categorical variables. Because sNfL levels were not normally distributed, they were log-transformed. The relationship between sNfL and eGFR levels was assessed using unadjusted and multivariable adjusted weighted linear regression models to obtain the β, confidence intervals, and *p*-value in the crude model, models I and II. The weights were incorporated into the analysis itself. The Crude model was unadjusted; Model I was adjusted for gender, age, BMI; and Model II was adjusted for gender, age, BMI, ethnicity, alcohol consumption, smoking status, hypertension, diabetes, and CVD. To evaluate the stability of the results, eGFR was treated as both a continuous and a categorical variable, the latter categorized into three groups, across the three models. Additionally, trend tests were conducted using the median values of eGFR divided into three groups for further comparison. For further stability assessment, subgroup analyses were performed, calculating the β, confidence intervals, and *p*-value using weighted multivariate linear regression with adjustments based on Model II variables. Subgroup stratification factors included age, gender, BMI, smoking status, alcohol consumption, hypertension, diabetes, and CVD, as suggested by previous literature. The strata variable was not considered when stratifying by itself. Interaction analyses between the stratification variables and eGFR (per 10-unit) were also conducted. Restricted cubic spline (RCS) analysis and scatter plots were used to visualize the relationship between log sNfL and eGFR. Additionally, gender-specific RCS and scatter plots were generated to explore this relationship. Spearman correlation analysis was also conducted to describe the association between log sNfL and eGFR. Statistical analyses were carried out using R software (version 4.3.3), and a two-tailed *p* value of less than 0.05 was considered statistically significant.

## Results

### Baseline characteristics of population

**Figure 1:**
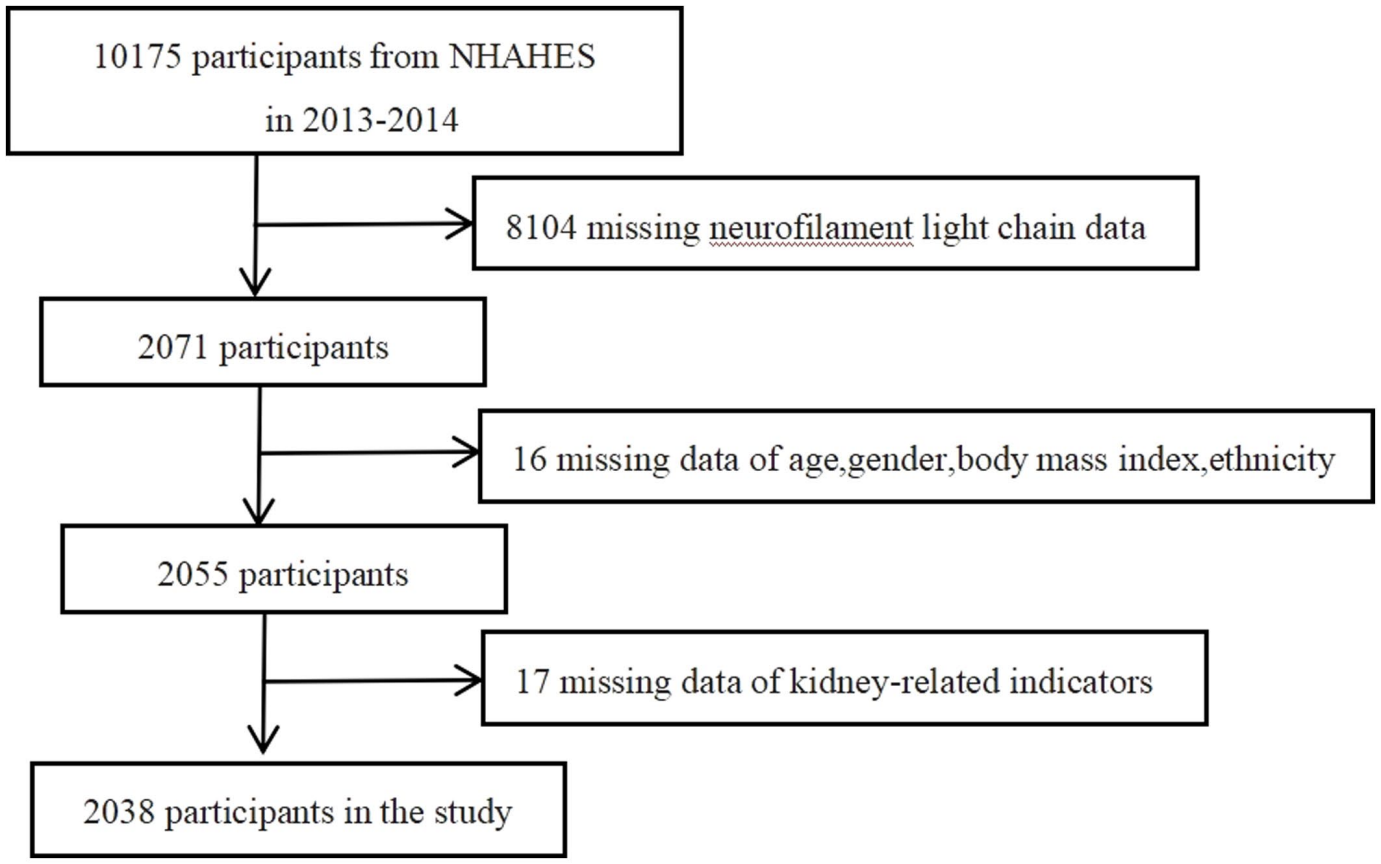
Flowchart for eligible participants selection.

In this study, a total of 2038 participants were included for final analysis based on the inclusion and exclusion criteria (Figure 1). Table 1 presented the baseline weighted characteristics of the enrolled population, stratified by tertiles of log sNfL. Males had higher sNfL levels than females in both the T2 and T3 groups (*p* < 0.01). Significant differences were observed among the three groups in terms of age, BMI, ethnicity, smoking status, hypertension, diabetes, and CVD (*p* < 0.05). As sNfL levels increased, eGFR decreased, while blood urea nitrogen, serum creatinine, and uric acid levels increased significantly (*p* < 0.01). The urine albumin-to-creatinine ratio was higher in the T3 group compared to the T1 and T2 groups (*p* < 0.01). However, no statistical differences were found in education, alcohol consumption, or urine creatinine (*p* > 0.05). Follow-up until December 31, 2019, revealed a weighted overall mortality rate of 3.1%. Weighted mortality rates were 1.1, 2.6, and 5.9% in the T1, T2, and T3 groups, respectively, indicating increased mortality with higher sNfL levels (*p* < 0.01).

**Table 1:** Characteristics of included participants based on log sNfL tertiles (*n* = 2038).

Characteristic	Overall	Log sNfL			*p*-value
		T1	T2	T3	
Gender, n (%)					0.010
Male	977 (48.8%)	283 (43.2%)	342 (51.9%)	352 (51.4%)	
Female	1,061 (51.2%)	394 (56.8%)	341 (48.1%)	326 (48.6%)	
Age(years), mean (SD)	44.91 (15.14)	35.15 (10.80)	46.44 (13.95)	53.82 (14.29)	<0.001
Body mass index, kg/m^2^, mean (SD)	29.30 (7.34)	29.56 (7.27)	28.49 (6.93)	29.92 (7.76)	0.008
Ethnicity, n (%)					0.001
Non-Hispanic white	892 (64.8%)	256 (54.8%)	308 (68.0%)	328 (72.1%)	
Non-Hispanic black	365 (12.0%)	122 (13.9%)	118 (11.3%)	125 (10.6%)	
Other	781 (23.2%)	299 (31.2%)	257 (20.6%)	225 (17.3%)	
Education, n (%)					0.4
Less than high school	444 (15.8%)	150 (17.6%)	135 (15.1%)	159 (14.6%)	
High school or above	1,594 (84.2%)	527 (82.4%)	548 (84.9%)	519 (85.4%)	
Alcohol consumption, n (%)					0.11
Light or less	867 (36.7%)	255 (33.5%)	296 (36.7%)	316 (40.0%)	
Moderate or more	1,171 (63.3%)	422 (66.5%)	387 (63.3%)	362 (60.0%)	
Smoking status, n (%)					0.020
Never smoker	1,146 (56.8%)	433 (64.0%)	373 (54.4%)	340 (51.6%)	
Former smoker	448 (22.1%)	110 (16.9%)	159 (23.3%)	179 (26.5%)	
Current smoker	444 (21.1%)	134 (19.1%)	151 (22.4%)	159 (22.0%)	
Hypertension, n (%)					<0.001
No	1,326 (68.0%)	543 (82.2%)	441 (65.8%)	342 (54.9%)	
Yes	712 (32.0%)	134 (17.8%)	242 (34.2%)	336 (45.1%)	
Diabetes, n (%)					<0.001
No	1,719 (87.7%)	638 (95.6%)	585 (88.3%)	496 (78.3%)	
Yes	319 (12.3%)	39 (4.4%)	98 (11.7%)	182 (21.7%)	
CVD, n (%)					<0.001
No	1,898 (94.0%)	670 (99.3%)	644 (94.5%)	584 (87.6%)	
Yes	140 (6.0%)	7 (0.7%)	39 (5.5%)	94 (12.4%)	
eGFR, ml/min/1.73 m^2^, median (IQR)	99.52 (84.85, 112.72)	110.58 (99.24, 119.68)	97.50 (85.13, 109.24)	89.50 (74.91, 101.61)	<0.001
eGFR, n (%)					<0.001
Low	87 (3.4%)	0 (0.0%)	14 (1.4%)	73 (9.2%)	
Medium	573 (28.8%)	93 (15.0%)	204 (30.9%)	276 (41.6%)	
High	1,378 (67.8%)	584 (85.0%)	465 (67.7%)	329 (49.2%)	
Blood urea nitrogen (mmol/L), mean (SD)	4.57 (1.76)	4.05 (1.30)	4.58 (1.42)	5.13 (2.27)	<0.001
Creatinine (umol/L), mean (SD)	77.23 (23.68)	71.56 (15.08)	76.68 (16.55)	83.97 (33.98)	<0.001
Uric acid (umol/L), mean (SD)	323.59 (83.08)	314.14 (77.48)	320.35 (83.82)	337.36 (86.42)	0.008
Urine albumin,(mg/L), median (IQR)	7.40 (4.10, 14.90)	8.00 (4.20, 15.80)	6.40 (3.90, 12.37)	8.20 (4.50, 17.29)	0.003
Urine creatinine (mmol/L), median (IQR)	9.46 (5.57, 14.67)	10.17 (5.66, 15.51)	8.84 (5.30, 14.16)	9.37 (5.86, 14.23)	0.094
UACR, (mg/g), median (IQR)	6.73 (4.62, 12.23)	6.52 (4.25, 11.43)	6.42 (4.62, 10.91)	7.89 (5.00, 15.13)	0.004
Survival condition					0.003
Survival	1,963 (96.9%)	669 (98.9%)	663 (97.4%)	631 (94.1%)	
Dead	75 (3.1%)	8 (1.1%)	20 (2.6%)	47 (5.9%)	

sNfL, serum neurofilament light chain; eGFR, estimated glomerular filtration rate; SD, standard deviation; IQR, interquartile range; CVD, cardiovascular and cerebrovascular related diseases; UACR, urine albumin-to-creatinine Ratio.

### Weighted linear regression analysis of association between sNfL and eGFR

When eGFR was analyzed as a continuous variable, a negative correlation with sNfL was observed. In the Crude Model, Model I, and Model II, each 10-unit increase in eGFR was significantly associated with reductions in log sNfL by −0.15 (−0.17, −0.13), −0.07 (−0.10, −0.05), and − 0.07 (−0.10, −0.04), respectively (Table 2). Furthermore, this inverse relationship persisted even when eGFR was categorized into three groups (Low, Medium, and High). After multivariate adjustments for age, gender, BMI, ethnicity, smoking status, alcohol consumption, hypertension, diabetes, and CVD, participants with the highest eGFR levels had significantly lower log sNfL levels by −0.46 (−0.89, −0.03) compared to those with the lowest eGFR levels (Table 2). Trend tests showed significant differences across the Crude Model, Model I, and Model II when eGFR was treated as a categorical variable (*p* < 0.001).

**Table 2:** Weighted liner regression analysis of association between Log sNfL and eGFR (*n* = 2038).

eGFR level	Crude Model β (95%CI)	*p*	Model I β (95%CI)	*p*	Model II β (95%CI)	*p*
Continuous variable (Per 10 units)	−0.15 (−0.17, −0.13)	<0.001	−0.07 (−0.10, −0.05)	<0.001	−0.07 (−0.10, −0.04)	<0.001
Categorical variable		<0.001		<0.001		<0.001
Low	Reference		Reference		Reference	
Medium	−0.59 (−0.81, −0.37)	<0.001	−0.42 (−0.68, −0.16)	0.005	−0.34 (−0.82, 0.14)	0.092
High	−0.96 (−1.2, −0.77)	<0.001	−0.54 (−0.78, −0.30)	<0.001	−0.46 (−0.89, −0.03)	0.045
*p* for trend		<0.001		<0.001		<0.001

Crude model: unadjusted; Model I: adjusted for gender, age, BMI; Model II: adjusted for gender, age, BMI, ethnicity, alcohol consumption, smoking status, hypertension, diabetes, and CVD. sNfL, serum neurofilament light chain; eGFR, estimated glomerular filtration rate; BMI, body mass index; CVD, cardiovascular and cerebrovascular related diseases; CI, confidence interval; β, regression coefficient.

### Stratified analyses of association between sNfL and eGFR

To further evaluate the robustness of the negative association between sNfL and eGFR, subgroup analyses were conducted, stratified by age, gender, BMI, smoking status, alcohol consumption, hypertension, diabetes, and CVD. Significant negative correlations between log sNfL and eGFR (per 10-unit increase) were observed across all subgroups (*p* < 0.01). Interaction analysis revealed a significant interaction between gender and eGFR (*p* = 0.032). Specifically, for each 10-unit increase in eGFR, log sNfL decreased by −0.06 (−0.09, −0.04) in males and by −0.07 (−0.11, −0.04) in females, with females demonstrating a slightly greater decrease in log sNfL. These findings are summarized in Table 3.

**Table 3:** Stratified analyses of association between Log sNfL and eGFR^#^ (*n* = 2038).

Variables	Model II β (95%CI)	*p*-value	interaction of β (95%CI), *p*-value
Stratified by gender			−0.02 (−0.05, 0.00), 0.032
Male	−0.06 (−0.09, −0.04)	<0.001	
Female	−0.07 (−0.11, −0.04)	<0.001	
Stratified by age			0.00 (0.00, 0.00), 0.13
<60 years	−0.10 (−0.13, −0.07)	<0.001	
≥60 years	−0.10 (−0.15, −0.06)	<0.001	
Stratified by body mass index			0.00 (0.00, 0.00), 0.8
<25 kg/m^2^	−0.07 (−0.11, −0.02)	<0.001	
25–30 kg/m^2^	−0.06 (−0.13, 0.00)	0.006	
>30 kg/m^2^	−0.07 (−0.12, −0.02)	<0.001	
Stratified by alcohol			0.02 (−0.03, 0.06), 0.3
Light or less	−0.09 (−0.14, −0.04)	<0.001	
Moderate or more	−0.05 (−0.08, −0.03)	<0.001	
Stratified by smoke			−0.01 (−0.04, 0.03), 0.6
Never smoker	−0.06 (−0.09, −0.03)	<0.001	
Former smoker	−0.09 (−0.14, −0.04)	<0.001	
Current smoker	−0.07 (−0.13, −0.01)	0.002	
Stratified by hypertension			−0.02 (−0.06, 0.01),0.055
No	−0.05 (−0.08, −0.02)	<0.001	
Yes	−0.10 (−0.14, −0.05)	<0.001	
Stratified by diabetes			−0.01 (−0.08, 0.06), 0.6
No	−0.06 (−0.08, −0.03)	<0.001	
Yes	−0.11 (−0.17, −0.04)	<0.001	
Stratified by CVD			−0.02 (−0.10, 0.06), 0.5
No	−0.06 (−0.09, −0.04)	<0.001	
Yes	−0.15 (−0.26, −0.05)	<0.001	

Model II: adjusted for gender, age, BMI, ethnicity, alcohol consumption, smoking status, hypertension, diabetes, and CVD. sNfL, serum neurofilament light chain; eGFR, estimated glomerular filtration rate; eGFR^#^: per 10-unit eGFR; BMI, body mass index; CVD, cardiovascular and cerebrovascular related diseases; CI, confidence interval; β, regression coefficient.

### The restricted cubic spline and scatter plot between log sNfL and eGFR

**Figure 2:**
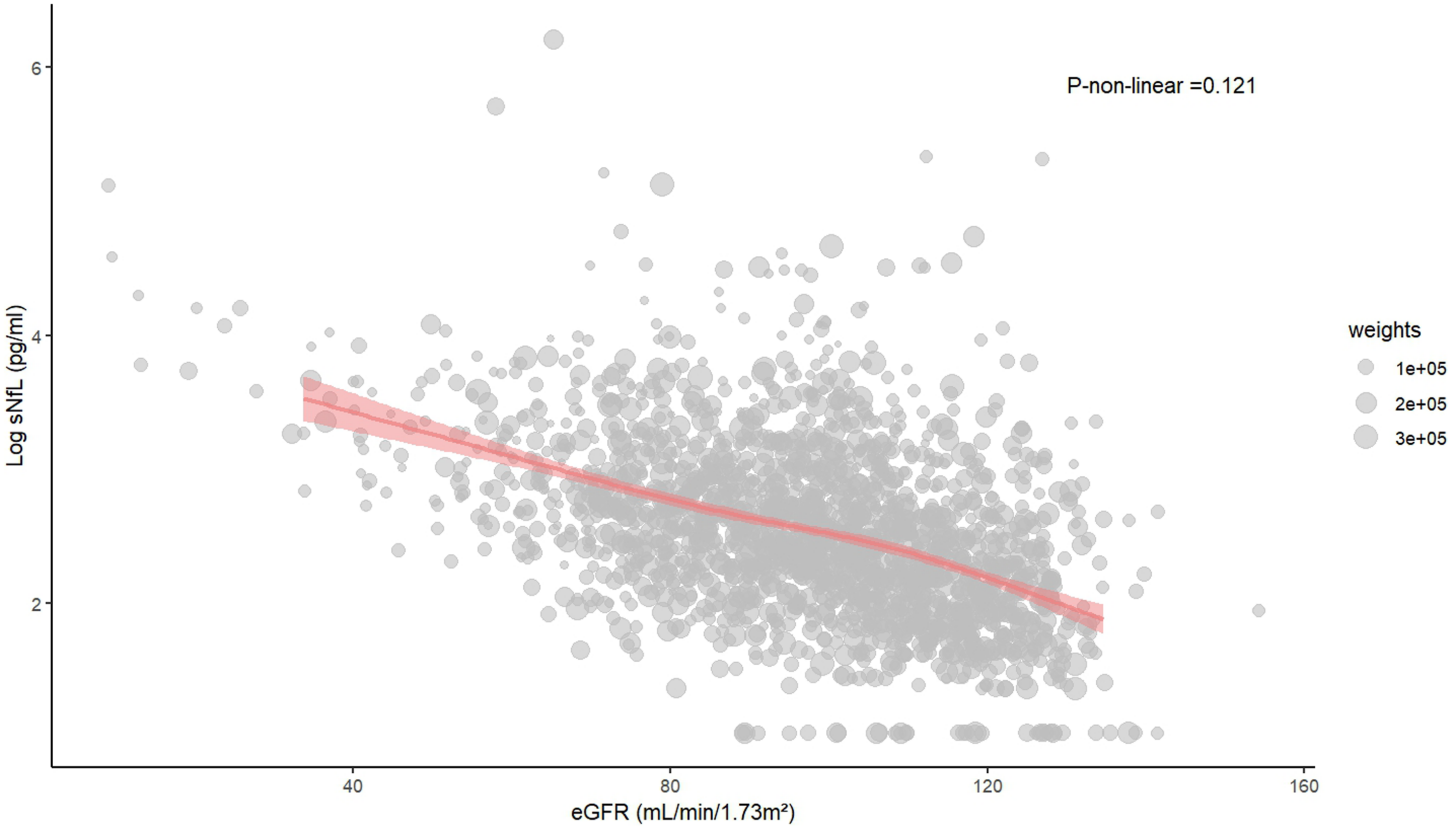
The restricted cubic spline and scatter plot between log sNfL and eGFR. sNfL, Serum neurofilament light chain; eGFR, estimated glomerular filtration rate.

**Figure 3:**
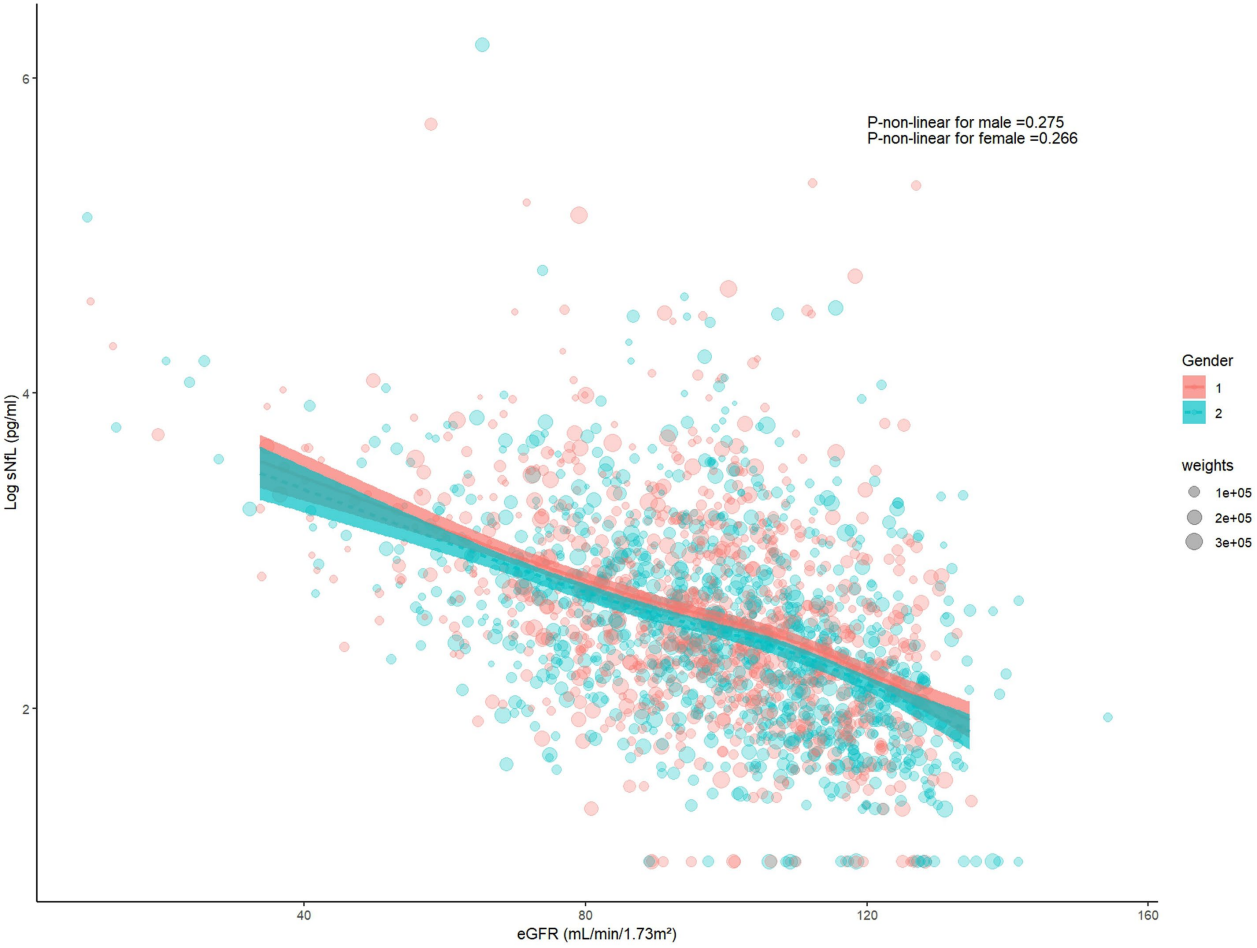
The restricted cubic spline and scatter plot between log sNfL and eGFR among males and females. (Gender = 1 represents males, Gender = 2 represents females). sNfL, Serum neurofilament light chain; eGFR, estimated glomerular filtration rate.

The restricted cubic spline indicated a linear relationship between log sNfL and eGFR (*p-*non-linear = 0.121). The Spearman correlation coefficient was −0.45 (*p* < 0.01). As eGFR increased, log sNfL levels gradually decreased (Figure 2). Further stratification by gender showed that both males and females exhibited decreasing log sNfL levels with higher eGFR levels. The curve revealed that females had slightly lower log sNfL levels compared to males at equivalent eGFR levels (Figure 3). The Spearman correlation coefficients were − 0.41 for males and − 0.49 for females (both *p* < 0.01).

## Discussion

Our study uncovered three key findings. Firstly, a significant negative correlation between log sNfL and eGFR levels was identified, which remained robust after multivariate adjustment and subgroup analysis. Secondly, interaction analysis highlighted a significant interaction between gender and eGFR levels. Females showed lower sNfL levels compared to males at equivalent eGFR levels, and the decrease in log sNfL with each 10-unit increase in eGFR was more pronounced in females. Lastly, the mortality rate was found to increase with higher sNfL levels. These findings deepen our understanding of kidney-brain interactions and underscore the potential impact of gender.

The relationship between kidney function and sNfL levels has been explored in several studies. Dittrich et al. studied 744 participants aged 70 years and older, adjusting for various confounding factors such as age, gender, hypertension, diabetes, smoking, and BMI. Their regression analysis revealed a statistically significant association between sNfL and CKD, as well as a significant correlation between sNfL and eGFR. Compared to participants with normal kidney function, those with CKD exhibited a higher linear regression coefficient between eGFR and sNfL (19). Akamine et al. studied 43 healthy participants aged 60 years and older, along with 188 diabetic patients, finding positive correlations between sNfL and serum creatinine levels, and negative correlations with eGFR levels (20). Kortvelyessy et al. also observed a positive correlation between sNfL and serum creatinine among 48 coronavirus infected patients, which was associated with adverse outcomes (21). In a prospective population-based cohort study with 17 years follow-up, it was found that declining kidney function was associated with increased levels of blood biomarkers related to dementia (such as sNfL and p-tau181), although not directly linked to dementia risk (22). Previous studies have shown a negative correlation between sNfL and eGFR levels, but these studies often involved older participants, small sample sizes, or were disease-specific, limiting the generalizability of their findings to broader populations. In contrast, our study utilized data from 2,038 participants in the NHANES database, covering individuals aged 20 years and older with an average age of 44.91 years. By rigorously adjusting for confounding factors observed in previous research, our study provides robust and widely applicable conclusions.

The mechanisms underlying how kidney function influences sNfL levels remain unclear. One hypothesis suggests that sNfL may be cleared by the kidneys, potentially leading to an overestimation of neuronal damage risk among individuals with impaired kidney function (22). Another hypothesis suggests that declining kidney function may contribute to neuronal damage through several pathways. Firstly, reduced levels of neuroprotective factors such as erythropoietin and active vitamin D have been implicated (23, 24). Secondly, elevated urea levels have been shown to alter the cytoskeleton and tight junction proteins in cultured endothelial cells, potentially disrupting the blood–brain barrier and increasing susceptibility to cerebral microbleeds (25). Additionally, cerebral artery mean flow velocity decreases over time during dialysis (26). Moreover, excessive activation of the renin-angiotensin-aldosterone system due to inflammation and oxidative stress may lead to sympathetic over activity, influencing blood pressure fluctuations and fluid balance, thereby impacting cerebral blood flow and neuronal health (27). These findings highlight the need for further investigation into the causal relationships between kidney and cerebral function through clinical and basic research efforts.

Notably, in this study, we observed inconsistent increases in sNfL levels at equivalent eGFR levels between genders. Specifically, females exhibited significantly lower sNfL levels than males at the same eGFR levels. Moreover, with each 10-unit increase in eGFR, the decrease in log sNfL was more pronounced in females. This phenomenon may be attributed to physiological and metabolic differences between genders, reflecting distinct roles of gender in kidney and nervous system injury and repair mechanisms. Research has shown that female estrogen receptor knockout mice exhibited aggravated damage in kidney ischemia–reperfusion injury, whereas female mice pre-treated with estrogen showed protective effects (28). Additionally, estrogen exerted protective effects by counteracting inflammation and mitochondrial dysfunction via the estrogen receptor α/silent mating type information regulation 2 homolog 1 pathway (29). In contrast, testosterone inhibited the activation of nitric oxide synthases and Akt following ischemia, as well as reduced the ratio of phosphorylated extracellular signal-related kinase to c-jun N-terminal kinase through non-androgen receptor mechanisms, leading to increased inflammation and heightened susceptibility to ischemia–reperfusion injury (30). Researches found that compared to control groups, ovariectomized female forebrain neuron-specific aromatase knockout mice exhibited diminished astrocyte activation, reduced hippocampal 17β-Estradiol levels, and more severe neuronal damage after global cerebral ischemia (31). Moreover, the study by Morale et al. demonstrated that estrogen protected midbrain dopaminergic neurons by regulating neuroglial inflammatory responses (32). These findings indicated that estrogen exerted protective effects in females by promoting anti-inflammatory and repair pathways, whereas testosterone in males might have enhanced inflammatory responses and susceptibility to damage, supported our study’s conclusions. This discovery has significant implications for further understanding kidney-brain interactions and highlights the importance of considering gender differences in the assessment and management of kidney-brain diseases in clinical practice.

Additionally, in this study, we found that elevated sNfL levels were significantly associated with increased mortality, which was consistent with previous findings. Nguyen et al. conducted a 14–15 year follow-up study on 294 African Americans aged 49–65 years, finding that baseline sNfL levels were significantly elevated in those who died (33). Rubsamen et al. studied 385 participants aged 65 years and older with no apparent neurological disorders, finding that sNfL was independently associated with all-cause mortality (34). The participants in these studies were relatively older, necessitating caution when generalizing these conclusions to younger populations. However, recent studies on populations aged 20 years and above have found a significant positive correlation between higher sNfL levels and all-cause mortality (35, 36). Similar to our study, but with inconsistent inclusion exclusion criteria, we demonstrated that the efficacy of sNfL in predicting death outcomes was stable. These findings indicated that sNfL not only reflects the health status of the nervous system but can also predict overall health and survival outcomes.

Our study possessed several notable strengths. First, we utilized complete data from the 2013–2014 NHANES database, which included a broad and representative sample of participants, including younger individuals. By conducting weighted analyses based on NHANES sampling characteristics, our study results demonstrated high external validity and generalizability. Second, we employed multivariable adjustments and subgroup analyses in our evaluation. These adjustments not only helped eliminate the influence of confounding factors but also allowed for a robust assessment of the independent relationship between eGFR and sNfL, thereby enhancing the internal validity of our findings. Third, the database utilized standardized methods for data collection and uniform sNfL measurement techniques, ensuring the accuracy and reliability of the data.

However, despite these strengths, our study had some limitations that needed to be acknowledged. Although our study sample was large and representative, the relationship between sNfL and eGFR was primarily based on cross-sectional statistical analysis, which prevented us from establishing a definitive causal relationship. Additionally, the 2013–2014 cycle of the NHANES database did not measure cerebrospinal fluid (CSF) NfL levels, leaving the relationship between CSF NfL levels and kidney function unclear. Nevertheless, previous literature reported a strong correlation between sNfL levels and CSF NfL levels (37, 38). Finally, although we adjusted for multiple variables, the possibility of residual confounding could not be completely ruled out.

In summary, this study revealed a significant negative correlation between sNfL and eGFR levels and identified potential gender differences in this relationship. Our findings offered a new perspective on understanding the intricate connections between kidney function and brain health. However, the causal relationship between kidney-brain interaction and gender disparities require further longitudinal and mechanistic studies to validate these findings.

## Data availability statement

The raw data supporting the conclusions of this article will be made available by the authors, without undue reservation.

## Ethics statement

The studies involving humans were approved by National Center for Health Statistics Research Ethics Review Board. The studies were conducted in accordance with the local legislation and institutional requirements. The participants provided their written informed consent to participate in this study.

## Author contributions

HP: Data curation, Writing – original draft, Methodology. ZL: Methodology, Writing – original draft. BH: Formal analysis, Software, Writing – review & editing. SZ: Writing – review & editing. YY: Writing – original draft.

## Footnotes

^1^ https://wwwn.cdc.gov/Nchs/Nhanes/2013-2014/SSSNFL_H.htm

^2^ https://wwwn.cdc.gov/nchs/nhanes/analyticguidelines.aspx
